# The evolution of the histone methyltransferase gene *Su(var)3-9 *in metazoans includes a fusion with and a re-fission from a functionally unrelated gene

**DOI:** 10.1186/1471-2148-6-18

**Published:** 2006-03-02

**Authors:** Veiko Krauss, Anne Fassl, Petra Fiebig, Ina Patties, Heinz Sass

**Affiliations:** 1 Department of Genetics, Institute of Biology II, University of Leipzig, Johannisallee 21–23, 04103 Leipzig, Germany

## Abstract

**Background:**

In eukaryotes, histone H3 lysine 9 (H3K9) methylation is a common mechanism involved in gene silencing and the establishment of heterochromatin. The loci of the major heterochromatic H3K9 methyltransferase Su(var)3-9 and the functionally unrelated γ subunit of the translation initiation factor eIF2 are fused in *Drosophila melanogaster*. Here we examined the phylogenetic distribution of this unusual gene fusion and the molecular evolution of the H3K9 HMTase Su(var)3-9.

**Results:**

We show that the gene fusion had taken place in the ancestral line of winged insects and silverfishs (Dicondylia) about 400 million years ago. We cloned *Su(var)3-9 *genes from a collembolan and a spider where both genes ancestrally exist as independent transcription units. In contrast, we found a *Su(var)3-9*-specific exon inside the conserved intron position 81-1 of the *eIF2γ *gene structure in species of eight different insect orders. Intriguinly, in the pea aphid *Acyrthosiphon pisum*, we detected only sequence remains of this *Su(var)3-9 *exon in the *eIF2γ *intron, along with an *eIF2γ*-independent *Su(var)3-9 *gene. This reveals an evolutionary re-fission of both genes in aphids. Su(var)3-9 chromo domains are similar to HP1 chromo domains, which points to a potential binding activity to methylated K9 of histone H3. SET domain comparisons suggest a weaker methyltransferase activity of Su(var)3-9 in comparison to other H3K9 HMTases. Astonishingly, 11 of 19 previously described, deleterious amino acid substitutions found in Drosophila Su(var)3-9 are seemingly compensable through accompanying substitutions during evolution.

**Conclusion:**

Examination of the *Su(var)3-9 *evolution revealed strong evidence for the establishment of the *Su(var)3-9*/*eIF2γ *gene fusion in an ancestor of dicondylic insects and a re-fission of this fusion during the evolution of aphids. Our comparison of 65 selected chromo domains and 93 selected SET domains from Su(var)3-9 and related proteins offers functional predictions concerning both domains in Su(var)3-9 proteins.

## Background

Heterochromatin typically represents a large differentiated chromatin compartment within eukaryotic nuclei (reviewed in [1]). While the mechanisms involved in molecular definition of facultative heterochromatin seem diverse [2,3], the central role of histone H3 lysine 9 di- and trimethylation (H3K9me2 and H3K9me3) for the establishment of constitutive heterochromatin and heterochromatic gene silencing has been shown for Drosophila, Schizosaccharomyces and Arabidopsis [4-7]. The majority of this heterochromatic H3K9 methylation is mediated by histone methyltransferases (HMTases) of the Su(var)3-9 family (for review see [8]). While the phylogenetic distribution of the Su(var)3-9-linked silencing pathway seems fairly broad, we note that so far all examined organisms have pericentromeric heterochromatin, which covers the main chromosomal distribution of the Su(var)3-9 protein in Drosophila [4].

**Figure 1:**
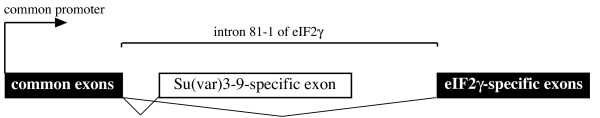
**The Su(var)3-9/eIF2γ gene fusion as it was previously found in several holometabolic insects [10]. **In this schematic drawing, a Su(var)3-9-specific exon is shown inside the conserved eIF2γ intron 81-1, which is situated after the first nucleotide of the *D. melanogaster*-codon 81 of eIF2γ. This gene structure expresses two mRNA variants, which encode two functionally unrelated proteins, through 3'alternative splicing from a common promoter and was detected in several species of flies, butterflies and beetles.

The Su(var)3-9 encoding gene of *Drosophila melanogaster*, originally isolated as dominant suppressor of position-effect variegation (PEV), expresses two distinct mRNA classes of 2.0 and 2.4 kb [9]. These mRNA variants emerge via 3' alternative splicing and have the first 80 amino acid residues of their open reading frames in common (Fig. 1). The molecular analysis of mutants showed that the PEV suppressor function is exclusively connected with the 2.4 kb mRNA which encodes Su(var)3-9 [9]. In contrast, the 2.0 kb mRNA codes for the γ subunit of the eukaryotic translation initiation factor 2 (eIF2γ) [10].

In known non-insect genomes, both *Su(var)3-9 *and *eIF2γ *are independent genes. Su(var)3-9 proteins bind to chromosomes in the nucleus, whereas eIF2γ proteins are cytoplasmic and interact temporary with ribosomes. Su(var)3-9 orthologues are histone H3K9-specific methyltransferases. In contrast, eIF2γ is a subunit of a G protein that delivers the methionyl initiator tRNA to the small ribosomal subunit and releases it upon GTP hydrolysis following the recognition of the initiation codon (for review see [11]). Despite their disparity in function and evolution, the Drosophila Genome Database [12] classified the two proteins as derived from a single gene.

Here we studied Su(var)3-9 orthologues, mainly of arthropods, to understand the following issues of interest: (1) How far is the *Su(var)3-9/eIF2γ *gene fusion distributed phylogenetically? (2) How stable is this gene arrangement in evolution? (3) Do Su(var)3-9 HMTases also exist in species with holocentric chromosomes (for e.g. earwigs, hemipterids, butterflies), which do not contain pericentromeric heterochromatin? (4) How do the amino acid substitutions of Drosophila Su(var)3-9 mutant proteins lead to deleterious phenotypes?

Here we show that the natural gene fusion of *Su(var)3-9 *and *eIF2γ *had taken place in the ancestral line of winged insects and silverfishs about 400 million years ago. We cloned *Su(var)3-9 *from a collembolan and a spider where both genes ancestrally exist as independent transcription units. In contrast, we found a *Su(var)3-9*-specific exon inside the conserved intron position 81-1 of the *eIF2γ *gene structure in more than a dozen insect species which are members of eight different orders. However, the aphid *Aphis sambuci *does not contain a Su(var)3-9-specific exon at any position of the otherwise conserved *eIF2γ *gene structure. In the pea aphid *Acyrthosiphon pisum*, we identified non-functional remains of this *Su(var)3-9 *exon in the *eIF2γ *intron, along with a novel, *eIF2γ*-independent *Su(var)3-9 *gene copy. These pieces of evidence demonstrate an evolutionary re-fission of *Su(var)3-9 *from *eIF2γ *in aphids. Furthermore, we explored the phylogenetic distribution of Su(var)3-9-orthologous proteins by bioinformatic means and used the identified Su(var)3-9 protein sequences for phylogenetic analysis to uncover times of Suv39h duplications occurred during the evolution of vertebrates. Su(var)3-9 chromo and SET domains were compared to exploit evolutionary and mutant substitutions for the prediction of functional roles of distinct protein regions.

## Results

### *Su(var)3-9 *gene identification, alignment and phylogenetic analysis

We cloned *Su(var)3-9 *cDNAs from ten distantly related arthropod species, which are *Araneus diadematus *(spider), *Allacma fusca *(springtail), *Lepisma saccharina *(silverfish), *Enallagma cyathigerum *(damselfly), *Forficula auricularia *(earwig), *Acyrthosiphon pisum *(aphid), *Cercopis vulnerata *(cicada), *Apis mellifera *(honey bee), *Bombyx mori *(silk worm) and *Drosophila nasutoides *[see Additional file 1]. Additionally, *Su(var)3-9 *sequences of 18 metazoan species, including 9 further arthropods, were retrieved by database mining. Eight other *Su(var)3-9 *orthologues have been described previously [9,10,13,14]. With the exception of Takifugu, Tetraodon, Oryzias (all fishes), Tribolium (red flour beetle) and most Drosophila species, all *Su(var)3-9 *genes are proved to be transcribed (Tab. 1). By analysis of recent genome projects we found that the Su(var)3-9 gene is a single copy gene, except in most vertebrates and some nematodes. All nematode sequences were excluded from the sequence set, because the corresponding *Su(var)3-9*-like ORFs showed highly divergent sequences. We also excluded all mammalian sequences other than Homo and Mus, because protein identity between these species is exceedingly high. In total, this led to the selection of 40 complete sequences in 36 species (Tab. 1).

**Figure 2:**
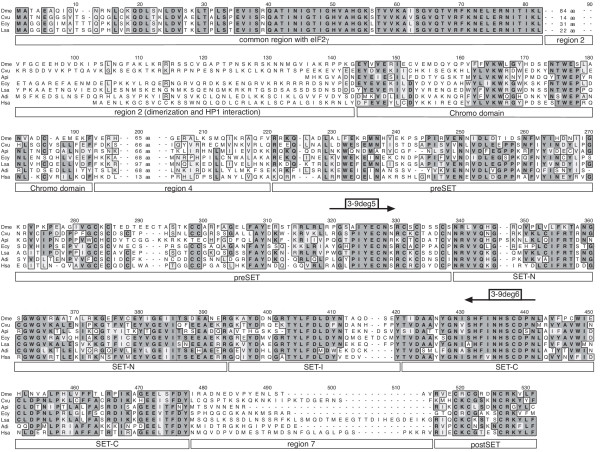
**Alignment of selected Su(var)3-9 proteins. **Arthropod proteins with and without N-terminal eIF2γ fusion are represented by *Drosophila melanogaster *(Dme) [9], *Cercopis vulnerata *(Cvu, cicada), *Acyrthosiphon pisum *(Api, aphid), *Enallagma cyathigerum *(Ecy, damselfly), *Lepisma saccharina *(Lsa, silverfish) and *Araneus diadematus *(Adi, spider). Additionally, human SUVH1 is shown (Hsa) [13]. Identical amino acid residues are underlaid dark gray, whereas similar residues are marked light gray. The domains of Su(var)3-9 are marked below the alignment. Binding sites of used degenerate oligonucleotide primers are shown above the sequences.

An alignment of these Su(var)3-9 proteins was obtained. During this step, the sequences from *Dictyostelium discoideum *and *Hydra magnipapillata *were excluded because these proteins do not contain a chromo domain. In Hydra, this is proven by a translation stop signal in frame with and upstream of a putative start AUG which is identically contained in three different ESTs. In the remaining alignment, positional identity was impossible to establish in areas located N-terminal of the chromo domain (Fig. 2). Therefore, these residues were truncated, leaving 545 alignment positions for analysis. The final Su(var)3-9 protein alignment [see Additional file 2] indicates that 21% of the included Su(var)3-9 amino acid residues are identical in 90% of the metazoan species studied. Moreover, 235 residues (43%) are identical in more than 50% of the proteins.

**Figure 3:**
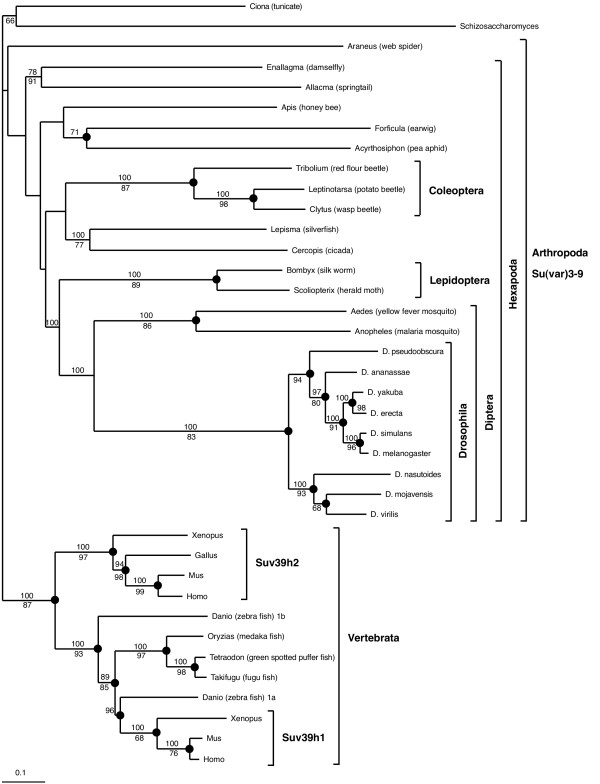
**Weighbor tree of Su(var)3-9-like proteins, with groups of interest highlighted. **The posterior probability (BI) is given above each supported node in percent of trees showing the same topology. The quartet-puzzling value (ML) is given below each supported node. If the same topology is also supported by MP analysis, the corresponding node is marked with a dot.

Phylogenetic analysis was carried out using this alignment including all Su(var)3-9 protein parts except the common region with eIF2γ and the region 2 (Fig. 2) [see Additional file 2] and implementing BI, ML, MP and weighbor methods (see Materials and Methods). The weighbor tree, which provides additional branching information from the other trees, is presented (Fig. 3). Monophyletic groups of Vertebrata, Coleoptera, Lepidoptera, Culicidae and Drosophila were strongly supported within all analyses. The groupings of Diptera and Mecopteroidea (Diptera+Lepidoptera) also gained remarkable support. However, other arthropod taxa (for e.g. Pterygota, Hexapoda and Arthropoda itself) were not consistently supported. Suv39h of the chordate Ciona branches as sister group of Vertebrata in BI and MP analyses only (data not shown). In spite of this, the tree dates the Suv39h gene duplication previously found in man and mouse [13] as occurred in the last common ancestor of tetrapodes. An independent duplication exists in *Danio rerio *(zebra fish), designated Suv39h1a and Suv39h1b by us, which cannot be found in other teleosts as Oryzias, Tetraodon and Takifugu. Topology and branch lengths reveal a slightly relaxed purifying evolution during the early divergence of the paralogous genes Suv39h2 and Suv39h1b.

### Phylogenetic mapping of the *Su(var)3-9/eIF2*γ gene fusion

**Figure 4:**
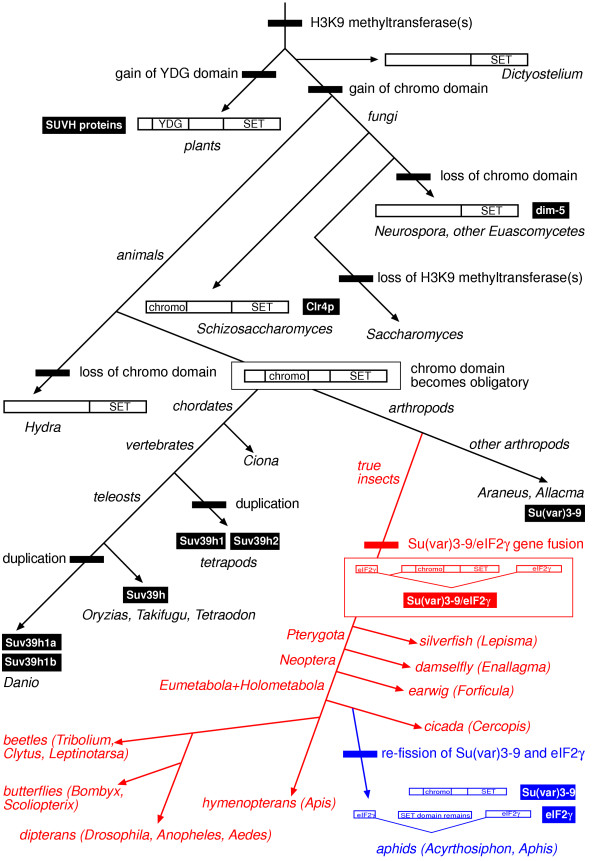
**Phylogenetic mapping of *Su(var)3-9 *evolutionary transitions. **Domain gains and losses, gene duplications and the fusion with as well as the re-fission from *eIF2γ *are shown. Commonly supported phylogeny were used, but for position of hymenopterans see Krauss et al. [19]. The branches of the tree are not to scale. All events are marked with bars, taxa names are italic and protein names are underlaid.

Next, we undertook a mapping of major evolutionary transitions of *Su(var)3-9 *genes and proteins using commonly accepted phylogenetic relationships (Fig. 4). Accordingly, Su(var)3-9-like H3K9 methyltransferases might have gained the chromo domain in the common ancestor of fungi and animals, because the Schizosaccharomyces Su(var)3-9 ortholog Clr4p also contains this domain. If this is correctly inferred, the chromo domain of Su(var)3-9-like proteins got lost at least two times independently during the evolution of ascomycetes (dim-5 proteins of Neurospora and five other species of Euascomycetes) and of cnidarians (Hydra). Alternatively, both bilaterian animals and an ancestor of fission yeast acquired chromo domains independently. Interestingly, plant SUVH H3K9 HMTases had gained a YDG domain instead of a chromo domain [15]. Like classical chromo domains [16], the YDG domain displays a strong interaction with the N-terminal tail of histone H3 [17] and might, thus, be convergent to Su(var)3-9 chromo domains.

In bilaterian animals, the chromo domain appears obligatory for Su(var)3-9 proteins. However, there is a great variability in length and sequence of the protein region N-terminal to the chromo domain. We found that, in true insects (Ectognatha), this protein region generally contains the N-terminus of the functionally unrelated γ subunit of the eukaryotic translation initiation factor2 (eIF2γ). Moreover, we detected that in twelve genera of insects, belonging to the eight different orders Zygentoma, Odonata, Dermaptera, Hemiptera, Hymenoptera, Coleoptera, Lepidoptera and Diptera, *Su(var)3-9 *and *eIF2γ *mRNAs have identical 5'ends, encoding a common N-terminus of both proteins that is between 79 to 84 amino acid residues long (Fig. 2, 4). In 20 out of 22 examined species of true insects, we gained evidence by genomic and/or cDNA sequence analysis that a *Su(var)3-9*-specific exon is located inside the intron 81-1 of *eIF2γ *. In each case, the expression of both gene products is regulated by alternative splicing. The common N-terminus of both proteins takes part in important functions inside eIF2γ proteins (for e.g. GTP binding) but does not constitute a closed globular domain [18]. In Drosophila Su(var)3-9, this common N-terminus might be involved in completely unrelated functions (see below). The totally different role of the common N-terminus in both proteins led us to conclude that the fusion of the *Su(var)3-9 *and the *eIF2γ *gene was a non-selected, accidental evolutionary event. Therefore, it should have occurred only once.

On the other hand, we cloned non-fused, complete *Su(var)3-9 *cDNAs and gene structures from *Araneus diadematus *(spider, Chelicerata) and *Allacma fusca *(springtail, Collembola). We noted further that the *eIF2γ *intron 81-1, containing the *Su(var)3-9*-specific exon under fused conditions, was found in all examined Hexapoda (including *Allacma fusca*, where it does not contain a *Su(var)3-9 *exon) and in *Oniscus asellus *(woodlouse, Crustacea) but not outside the Pancrustacea [19]. Thus, the *Su(var)3-9/eIF2γ *gene fusion has occurred in the common ancestor of winged insects and silverfishs after the branching of collembolans and may be a synapomorphy of the Dicondylia or the Ectognatha (Dicondylia+Archaeognatha).

**Figure 5:**
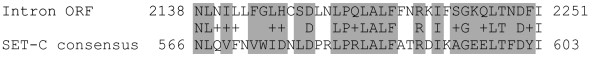
**Residual Su(var)3-9 ORF in the *eIF2γ *intron 81-1 of pea aphids. **This reading frame is located between nucleotide 2138 and 2251 of the 2539-nt-intron in the same orientation as the surrounding eIF2γ ORF. The lower sequence is a consensus of 38 Su(var)3-9 proteins in the corresponding region SET-C. Positions are given according to *D. melanogaster *Su(var)3-9. Identical and similar amino acids are marked. Residues found identically in the majority of Su(var)3-9 proteins are boxed grey.

Concomitantly, we got evidence that this gene fusion has become evolutionarily reverted at least once during the evolution of hemipterid insects. The complete *Su(var)3-9 *cDNA and gene structure from the pea aphid *Acyrthosiphon pisum *(Fig. 2, 4) was cloned. No similarities to *eIF2γ *sequences were found in this *Su(var)3-9 *gene, and the 81-1 introns of the *eIF2γ *genes of *Acyrthosiphon pisum *and *Aphis sambuci *do not contain a *Su(var)3-9*-specific exon. Most interestingly, whereas the Aphis intron does not show significant similarities to other Genbank sequences, the Acyrthosiphon intron reveals a residual similarity to Su(var)3-9 SET domain sequences (Fig. 5). In RT-PCR experiments, we did not detect any expression of this genomic region. We conclude that the fusion of *Su(var)3-9 *and *eIF2γ *established in true insects was re-fissioned in a common ancestor of Acyrthosiphon and Aphis.

Alternatively, the Acyrthosiphon *Su(var)3-9 *gene might represent an unfused paralog, which exists beside of the *Su(var)3-9/eIF2γ *gene fusion in all true insects. Three arguments render this hypothesis highly improbable. (1) We have cloned *Su(var)3-9 *independently of *eIF2γ *from five genera of insects (Lepisma, Enallagma, Cercopis, Clytus and Scoliopteryx) and identified the connection with *eIF2γ *in each case later. (2) We were unable to detect an additional, non-fused *Su(var)3-9 *gene in complete or almost complete sequenced genomes of six insect genera (Apis, Tribolium, Bombyx, Aedes, Anopheles and Drosophila). (3) Both introns found in the non-fused Acyrthosiphon *Su(var)3-9 *gene are novel, because all introns identified in other metazoan *Su(var)3-9 *genes including the collembolan *Allacma fusca *are located in non-homologous positions of the ORF (data not shown). Thus, it is supposed that the aphid gene has acquired these introns after the fission of *Su(var)3-9 *from an *eIF2γ *gene. Therefore, we summarize that *Su(var)3-9 *represents a gene which was fused with and re-fissioned from a functionally unrelated gene during the evolution of insects.

### Molecular evolution and functional aspects of Su(var)3-9 protein domains

The conservation of the chromo, preSET, SET and postSET regions shows that the corresponding domains has been subjected to strong purifying selection. In contrast, the regions 2, 4 and 7 of the Su(var)3-9 alignment (Fig. 2) reveal much less sequence conservation. Whereas region 4 and 7 do not associate with any known function, region 2 serves, possibly together with the common region of eIF2γ and Su(var)3-9, as dimerization domain in Drosophila and is involved in interaction with HP1 and Su(var)3-7 [4,20]. Thus, the *D. melanogaster *N-terminus is essential for full enzymatic activity, which is obtained through Su(var)3-9 dimerization, and correct nuclear localization, which is dependent on interactions with HP1 and Su(var)3-7 [4,21]. At least the HP1 interaction seems to be conserved in the N-terminus of the mammal Suv39h1 proteins [13], however, there cannot be found conserved amino acid residues N-terminal of the chromo domain between insect and vertebrate proteins (Fig. 2 and data not shown). A significant conservation of region 2 was identified only between Su(var)3-9 proteins found in species of the same insect order. The Predict Protein server [22] predicts highly diverse, loop-rich secondary structures in this region, and a SEG analysis [23] detected repetitive sequence elements in region 2 of 11 out of 16 arthropod genera. Thus, we argue that region 2 is only weakly evolutionarily constrained in insects. Moreover, Schizosaccharomyces Clr4p and Acyrthosiphon Su(var)3-9 contain only six or eight amino acid residues N-terminal to the chromo domain, respectively. Together, these pieces of evidence constrict the functional conservation between arthropod and chordate Su(var)3-9 proteins to chromo, preSET, SET and postSET regions, which does not exclude a random co-localization of functions in other regions of Su(var)3-9 orthologues.

**Figure 6:**
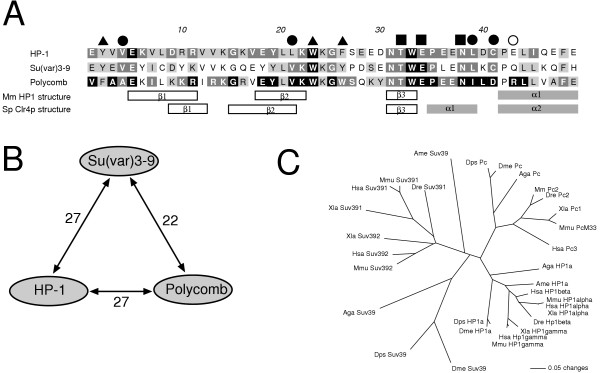
**Chromo domain comparison between selected Su(var)3-9, HP1 and Polycomb proteins. **A. Alignment of consensus sequences. Absolute identical residues are black boxed, 75–99%-identical ones are dark grey boxed and 74–50% identical amino acid positions are light grey boxed for each class of orthologues. Functionally important residues are marked by a filled triangle (methyl lysine recognition), a filled quad (histone H3K9/K27 side chain or backbone recognition) or a filled circle (recognition of the lysine 2 of the H3 peptide) according to Nielsen et al. [26]. An arginine (position 43 of the alignment, marked with an empty circle), which is specifically conserved in Polycomb proteins, is important for preference of H3K27me against H3K9me [27]. Determined secondary structures of HP1 [26] and Clr4p [52] are given beneath the alignment. B. Numbers of identical consensus residues between Su(var)3-9, HP1 and Polycomb chromo domains. C. Unrooted neighbour joining tree of selected Su(var)3-9, HP1 and Polycomb chromo domains from vertebrates and insects. Only species which have orthologues of all three proteins are included. Abbreviations: Aga, *Anopheles gambiae*; Ame, *Apis mellifera*; Dme, *Drosophila melanogaster*; Dps, *Drosophila pseudoobscura*; Dre, *Danio rerio*; Hsa, *Homo sapiens*; Mmu, *Mus musculus*; Xla, *Xenopus laevis*.

Chromo domains were detected in all bilaterian Su(var)3-9 proteins and in Clr4p of fission yeast (Fig. 2, 4). Functional analyses of diverse chromo domains revealed an unexpected diversity of interactions, including those with histone H3 tails, DNA and RNA (for review see [16]). Although the chromo domain is important for heterochromatic binding of Su(var)3-9 proteins in Schizosaccharomyces, mammals and Drosophila [4,24,25], the exact target molecule of the Su(var)3-9 chromo domain is unknown. Su(var)3-9 proteins contain a classical chromo domain [16], which is especially similar to HP1 and Polycomb chromo domains. Therefore, we have compared chromo domains of 34 Su(var)3-9, 21 HP1 and 10 Polycomb proteins (Fig. 6). We found that in three out of four conserved amino acid residues, which are essential for the specifity of HP1 binding to histone H3K9me [26], Su(var)3-9 proteins show the same conservation as HP1 proteins as well as a conserved difference to Polycomb proteins (Fig. 6A, filled circles). In contrast, an arginine at position 43 of the chromo domain alignment, which is necessary for interaction with histone H3K27me [27], is conserved only in Polycomb proteins (Fig. 6A, empty circle). Both results argue for a histone H3K9me preference of Su(var)3-9 chromo domains. Concomitantly, both alignment and tree branch lengths (Fig. 6A, C) infer a stronger chromo domain sequence conservation in HP1 than in Su(var)3-9 proteins. This might reflect a lesser affinity to histone H3 tails or a lesser discrimination between differently modificated H3K9 moieties, as compared to HP1 proteins. The latter opportunity was supported by pull-down assays using human SUV39H1 [28]. Thus, Su(var)3-9 proteins generally may be able to interact with histone H3 tails by chromo domains to facilitate and/or to locally restrict histone methylation. However, an interaction of the Su(var)3-9 chromo domain with heterochromatin-specific RNA cannot be excluded.

**Figure 7:**
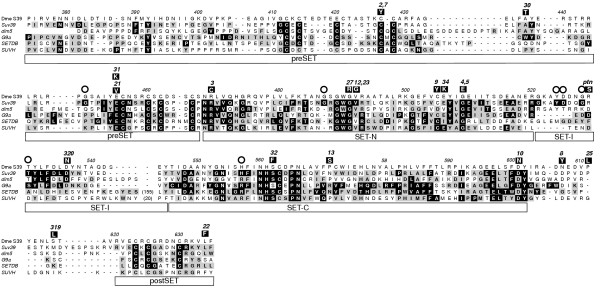
**Alignment of histone H3K9 methyltransferase consensus sequences that includes preSET, SET and postSET regions. **Alignment positions are given according to *D. melanogaster *Su(var)3-9. Specific consensus sequences were computed from 38 Su(var)3-9, 8 dim-5, 12 G9a/GLP, 15 SETDB/EuHMTase and 19 plant SUVH proteins. 90–100%-identical residues are black boxed and 50–89%-identical ones are grey boxed for each class of orthologues. Above the proteins, amino acid substitutions found in mutant *D. melanogaster *Su(var)3-9 proteins [6, 34] are black shadowed. Persistently conserved consensus differences found in Su(var)3-9 proteins in comparison to other H3K9 methyltransferases are marked with an empty circle.

The identification and characterization of point mutations in Su(var)3-9 of *Drosophila melanogaster *which lead to differential HMTase activities [6] demonstrated that the functional potential of Su(var)3-9 proteins is mainly determined by the kinetic properties of the HMTase reaction which is in turn dependent of SET- and SET-associated regions. We compared the structural conservation of these regions between Su(var)3-9 and other HMTases (dim-5, G9a/GLP, SETDB and SUVH proteins) which are proved to be histone H3K9 methylases [7,29-31]. Su(var)3-9 proteins contain only four persistent conserved sequence differences to these paralogous proteins: G450 (Fig. 7, position 450 according to *D. melanogaster *Su(var)3-9) in preSET, G487 in SET-N, YddqGrT (positions 524 to 530) in SET-I, and H557 in SET-C. We suggest that at least two of these conserved sequence deviations have interesting functional consequences.

First, whereas 36 out of 39 Su(var)3-9 orthologues contain a H557 in SET-C, 47 out of 54 examined non-Su(var)3-9 H3K9 HMTases contain an arginine at this position. The homologous R328 of Neurospora dim-5 is essential to permit trimethylation of histone H3K9 without intermediate release of H3K9-dim-5 binding, i.e. a processive type of reaction [32]. In dim-5, an arginine to histidine substitution at this position decreases the HMTase activity below 0.6 percent [33]. On the other hand, the activity of human SUV39H1 can be increased by a reciprocal exchange of histidine to arginine to more than 2000 percent [34]. It appears, therefore, that Su(var)3-9 proteins, in contrast to other H3K9 methyltransferases, are generally non-processive enzymes, as proved for *D. melanogaster *Su(var)3-9 [20]. In addition, Ebert et al. [6] identified the critical amino acid substitution (R529S) of the Drosophila gain-of-function mutant *Su(var)3-9*^*ptn *^within the above mentioned YDdqGrT motif. This gain-of-function mutant possesses a significantly increased HMTase activity that overcompensates amorphic mutations in Drosophila heterozygotes. In summary, two Su(var)3-9-diagnostic conservations of amino acids in the SET domain seem to suppress the HMTase activity of Su(var)3-9 proteins in comparison to other H3K9 methyltransferases. Furthermore, we found a conserved phenylalanine (Fig. 7, position 600), which supports that all Su(var)3-9 HMTases may be able to tri-methylate H3K9 [35].

Next, we analyzed 19 deleterious amino acid substitutions described for *D. melanogaster *Su(var)3-9 [6,36] using the H3K9 HMTase alignment (Fig. 7). We recognized that these substitutions fall into three classes (Tab. 2). Class I contains seven mutations exclusively found in totally conserved residues of preSET and SET-N regions of all H3K9 methylases. These substitutions typically show a null phenotype. The molecular functions of the corresponding residues can easily be drawn from structural analysis of Neurospora dim-5 or Schizosaccharomyces Clr4p [33,37] (Tab. 2). In contrast, class II contains three substitutions at positions which are conserved only in Drosophila, six other substitutions that were detected in some wild type Su(var)3-9 proteins of other species, one (C505Y) that was found similarly substituted (C505L) in the Su(var)3-9 protein of *Araneus diadematus*, and one (S562F) that was identified similarly substituted (S562L) in some G9a proteins. Class II substitution positions, thus, show only a partial conservation and became probably compensated through nearby substitutions which have been determined (see Materials and Methods, Tab. 2). The molecular function of these residues was commonly not revealed by comparison with structural analyses of homologous proteins. Class II substitutions were found in all SET domain-related regions and show hypomorphic or null phenotypes. *Su(var)3-9*^*ptn *^constitutes a third, hypermorphic class of mutants. Notably, we found an identical R529S substitution in several wild type Su(var)3-9 proteins of other species, but it seems that there does not exist a local compensatory amino acid substitution (Tab. 2). The R529S substitution may cause a higher HMTase activity of Su(var)3-9 proteins in those species, or become compensated by remote intramolecular changes.

Finally, we were specifically interested in the Su(var)3-9 sequence of *Drosophila nasutoides*. The *D. nasutoides *genome comprises 61,7% (female) or 67,4% (male) heterochromatin and represents by far the heterochromatin-richest genome of Drosophila [1]. However, we did not find any peculiarities in the *D. nasutoides *protein compared with other Su(var)3-9 sequences.

## Discussion

To our knowledge, fusions of two ancestrally independent genes with completely different functions similar to *Su(var)3-9/eIF2γ *have never been described so far. Other known gene fusions are supposed to be positively selected because the resulting gene products are fused players of the same cellular pathway, fused molecular interactors or perform at least one novel function using an acquired protein domain. How, then, was it possible that two proteins as different as Su(var)3-9 and eIF2γ in respect to sequence, structure, function, cellular localization and interactions were evolved to be derived from a single gene structure? Northern blots in Drosophila [9] revealed that the *eIF2γ *mRNA is expressed strongly in each developmental stage, whereas the *Su(var)3-9 *mRNA is expressed weakly during the first nine hours of embryonal development and almost undetectable during later stages. Therefore, we hypothesize that the *Su(var)3-9*-specific splice variant of the *Su(var)3-9/eIF2γ *gene "parasitize" on the strong expression of the *eIF2γ *splice variant. The developmental changes of the *Su(var)3-9 *share in the *Su(var)3-9/eIF2γ *primary transcript are unable to influence the *eIF2γ *expression significantly because of the generally weak expression rate of *Su(var)3-9*. Under these conditions, it was possible that a *Su(var)3-9 *retrotransposition into the 81-1 exon of an ancient *eIF2γ *gene has taken place and that this event has immediately resulted in a functional, alternative spliced gene. The only additional prerequisite is an activation of a cryptic splice site at the 5'end of the *Su(var)3-9*-specific exon, which has to be sufficient weak to not disturb significantly the *eIF2γ *expression.

To determine age and distribution of the *Su(var)3-9/eIF2γ *gene fusion, we have cloned orthologues of both genes or of the gene fusion, respectively, in 19 selected genera of arthropods ([10,19], this study). We found that the fusion is restricted to Ectognatha (Insecta) and, possibly, to Dicondylia (Pterygota + Zygentoma) (Fig. 4). According to palaeontological evidence with respect to the first true insect [38], the age of this unusual genomic assemblage can be estimated to about 400 million years. Irrespective of its long history, the gene fusion seems to impose a functional burden on the encoded gene products. In beetles and butterflies obvious splice artefacts, containing all exons of the fusion, are detectable [10]. The coding potential of these artefacts comprises all *eIF2γ *exons under inclusion of the *Su(var)3-9*-specific exon, which renders the encoded protein functionally inactive, at least with regard to eIF2γ. Notably, the *Su(var)3-9*-specific part of the gene fusion consists in all analyzed 21 species of only one large exon (>1450 bp). Initially, this may have been caused by retrotransposition of *Su(var)3-9 *sequences into the *eIF2γ *gene. Afterwards, the establishment of internal *Su(var)3-9 *introns might have been suppressed by selection against abundant functionless or antimorphic splice artefacts, which would concomitantly decrease the expression of functional *eIF2γ *mRNAs. At the same time, the *eIF2γ *part of the gene fusion has acquired at least four novel introns [19], and the newly emerged Acyrthosiphon *Su(var)3-9 *gene has gained two novel introns.

During this study, we found evidence for a reversion of the *Su(var)3-9/eIF2γ *gene fusion in aphids. The remnants of a Su(var)3-9-coding region in the *eIF2γ *intron 81-1 of *Acyrthosiphon pisum *reveal that these aphids descend from ancestors which harbored the gene fusion. Because the cicada *Cercopis vulnerata *possesses the fused gene, the fission of both gene parts has to be occurred during the evolution of the hemipterid group Sternorrhyncha (psyllids, whiteflies, aphids and coccids). It remains open whether a genomic duplication has happened, or a renewed retrotransposition of the *Su(var)3-9 *mRNA.

The central role of the Su(var)3-9 histone H3K9 methyltransferase for the establishment of pericentromeric heterochromatin has been shown for mammals, Drosophila and Schizosaccharomyces [4-6,28]. Our observation of Su(var)3-9 orthologues in holocentric species of insects (butterflies, hemipterans, earwigs) argues for an important role of the protein also outside of the pericentromeric heterochromatin, possibly in euchromatic gene silencing [24,39], at telomeres [40] and/or in chromosome segregation [41]. Whether Su(var)3-9 proteins are involved in the establishment of heterochromatic regions in aphid chromosomes, which are mostly limited to telomeres and X chromosomes ([42], and references therein), remains to be seen. Additionally, it would be interesting to evaluate function and nuclear distribution of a Su(var)3-9 ortholog in the coccid model system *Planococcus citri*, where H3K9 methylation is found exclusively in the paternally imprinted chromosome set [2].

## Conclusion

Our examination of the evolution of the *Su(var)3-9*/*eIF2γ *gene fusion revealed strong evidence for the establishment of this fusion in a common ancestor of dicondylic insects. Because of the unrelatedness of *Su(var)3-9 *and *eIF2γ *and the demonstrated broad phylogenetic distribution of the fusion, this gene structure is a reliable synapomorphy, but appears not to invoke novel functions of the gene products. Therefore, we interpret this gene fusion as an event of constructive neutral evolution as proposed by Stoltzfus [43]. The identified re-fission of this fusion during the evolution of aphids shows the vulnerability of this structure to evolutionary decay, probably due to duplication and partial degeneration.

Our comparison of chromo domains and SET domains from Su(var)3-9 and related proteins offers functional predictions concerning both domains in Su(var)3-9 proteins. Su(var)3-9 chromo domains are similar to HP1 chromo domains, which points to a potential binding activity to methylated K9 of histone H3. SET domain comparisons suggest less enzymatic activity of Su(var)3-9 proteins in comparison to other H3K9 HMTases. Su(var)3-9 proteins combine two motifs in one molecule, which are typical for structural (chromo domain) or enzymatic components (SET domain) of chromatin. This raises an interesting question: Are evolutionary attenuations of the chromo domain histone H3 binding affinity and of the SET domain histone H3 methyltransferase activity necessary conditions to make Su(var)3-9 compatible to animal chromatin? Domain swapping experiments may give an answer.

## Methods

### Sources of arthropods utilized

Species trapped in the vicinity of Leipzig (Sachsen, Germany) were *Araneus diadematus *(spider), *Lepisma saccharina *(silverfish), *Enallagma cyathigerum *(damselfly), *Forficula auricularia *(earwig), *Acyrthosiphon pisum *(aphid) and *Apis mellifera *(honey bee). *Cercopis vulnerata *(cicada) was captured around Ruhla (Thüringen, Germany). *Allacma fusca *(springtail) was trapped near Ilsenburg (Sachsen-Anhalt, Germany). *Bombyx mori *(silk worm) was used from commercial stock. DNA from *Drosophila nasutoides *was delivered by Dr. H. Zacharias (Kiel, Germany) as a courtesy.

### Isolation of *Su(var)3-9 *genes using PCR

DNA was isolated by standard protocols. Trizol reagent (Invitrogen) was used to isolate total RNA. cDNA was synthesized using Hminus-M-MLV reverse transcriptase (Fermentas) and a polyT primer. Degenerate primers based on the amino acid sequences of already known Su(var)3-9 proteins were designed to partially amplify the *Su(var)3-9 *gene from genomic DNA and/or cDNA of arthropod species. Used degenerated oligonucleotide primers were 3-9deg5 (5'-GCCHGGXRBXSCVATMTWYGARTGCAA-3') and 3-9deg6 (5'-GGATCRCAMGARTGRTTRATRAARTG-3'). Primer positions within *Su(var)3-9 *are shown in figure 2. PCR amplifications were done in a Gradient Cycler (Eppendorf) at annealing temperatures between 37°C and 62°C. The initial PCR product was purified using Spin PCRapid Kit (Macherey&Nagel) and sequenced. Species-specific primers were designed based on the received sequence to obtain 5'ends and 3'ends of *Su(var)3-9 *transcripts by 5'RACE (Rapid amplification of cDNA ends) and 3'RACE, respectively (GeneRacerKit, Invitrogen). Alternatively, we used *Su(var)3-9 *ESTs found in databases and already known sequences from the supposedly fused *eIF2γ *gene [19] for RACE experiments. Additionally, inverse PCR products from digested and ligated genomic DNA preparations were purified, cloned and sequenced. The specific sequencing strategy used for each of the analyzed species is given [see Additional file 1]. Species-specific primer sequences are listed [see Additional file 3].

Sequences were determined either by direct sequencing of the PCR fragment or by sequencing of two or three independent clones from different PCR reactions. PCR fragments were subcloned using pGEM-T PCR cloning kit (Promega). Sequencing was performed on ABI3100 equipment (ABI) using BigDye Sequencing Chemistry (ABI). For sequence analyses, MacVector 7.2 (Accelrys) was used.

### Sequence sampling and annotation

*Su(var)3-9*-orthologous DNA sequences from genome sequencing projects of metazoans were sampled from databases using BLAST. In particular, we used tBLASTn [44], based on seven already known Su(var)3-9 sequences [10], to retrieve *Su(var)3-9*-like genomic sequences from finished and unfinished genome projects deposited at the NCBI database. Additionally, single trace sequences were screened using discontiguous MEGABLAST and were assembled manually. Intron positions at the corresponding nucleotide sequences were deduced by co-occurrence of splice consensus sites and gaps in similarity. This exon-intron structure was confirmed by cDNA or EST sequences if available. The orthology of these candidate sequences was verified by reciprocal BLASTp analysis. We used only complete sequences which are orthologous to *Drosophila melanogaster *Su(var)3-9. A list of sequences and its sources is provided in table 1.

**Table 1:** List of *Su(var)3-9 *Gene Sequences used.

Species	Abbre- viation	Taxon^a^	Sequence determined by	Introns^b^	Expression	Genbank Accession Number
*Schizosaccharomyces pombe*	Spo	I	[24]	0	cDNA	AF061854
*Dictyostelium discoideum*	Ddi	II	genome project	?	EST	EAL72127
*Hydra magnipapillata*	Hma	III	genome project	2	EST	CN626687
*Homo sapiens*	Hsa1*	IVa	[13]	5	cDNA	BC006238
*Homo sapiens*	Hsa2*	IVa	genome project	5	cDNA	AK027067
*Mus musculus*	Mmu1*	IVa	[13]	5	cDNA	AF019969
*Mus musculus*	Mmu2*	IVa	[14]	5	cDNA	AF149205
*Gallus gallus*	Gga2*	IVb	genome project	5	cDNA	AJ851535
*Xenopus laevis*	Xla1*	IVc	genome project	5	cDNA	BC070805
*Xenopus laevis*	Xla2*	IVc	genome project	5	cDNA	CR926249
*Danio rerio*	Dre1a*	IVd	genome project	4	cDNA	BC052225
*Danio rerio*	Dre1b*	IVd	genome project	5	cDNA	BC076417
*Oryzias latipes*	Ola	IVd	genome project	4	n.d.	(golw_scaffold3430)
*Tetraodon nigroviridis*	Tni	IVd	genome project	4	n.d.	CAAE01010335
*Takifugu rubripes*	Tru	IVd	genome project	4	n.d.	CAAB01003782
*Ciona intestinalis*	Cin	V	genome project	7	cDNA	AK114564
*Araneus diadematus*	Adi	VIa	this study	0	RACE	AM050256
*Allacma fusca*	Afu	VIb	this study	6	RACE	AM050257, AM050258
*Lepisma saccharina*	Lsa	VIc	this study	?	RACE	AM050268
*Enallagma cyathigerum*	Ecy	VId	this study	0	RACE	AM050266
*Forficula auricularia*	Fau	VIe	this study	?	RACE	AM050267
*Acyrthosiphon pisum*	Api	VIf	this study	2	RACE	AM050261, AM050262
*Cercopis vulnerata*	Cvu	VIf	this study	0	RACE	AM050264
*Apis mellifera*	Ame	VIg	genome project, this study	0	RT-PCR	AADG04005784, AM050259
*Clytus arietis*	Car	VIh	[10]	0	RACE	AJ290961
*Leptinotarsa decemlineata*	Lde	VIh	[10]	0	RT-PCR	AJ290965
*Tribolium castaneum*	Tca	VIh	genome project	0	n.d.	(contig856)
*Scoliopterix libatrix*	Sli	VIi	[10]	0	RT-PCR	AJ290959
*Bombyx mori*	Bmo	VIi	genome project, this study	0	RT-PCR	AM050263
*Anopheles gambiae*	Aga	VIj	genome project	0	EST	AAB01008888
*Aedes aegypti*	Aae	VIj	genome project	0	EST	(trace)
*Drosophila melanogaster*	Dme	VIj	[9]	0	cDNA, RACE	AJ290956
*Drosophila simulans*	Dsi	VIj	genome project	0	n.d.	(trace)
*Drosophila yakuba*	Dya	VIj	genome project	0	n.d.	AAEU01000306
*Drosophila erecta*	Der	VIj	[10]	0	n.d.	AJ290957
*Drosophila ananassae*	Dan	VIj	genome project	0	n.d.	(trace)
*Drosophila pseudoobscura*	Dps	VIj	genome project	0	n.d.	AADE01000627
*Drosophila virilis*	Dvi	VIj	genome project	0	n.d.	(trace)
*Drosophila mojavensis*	Dmo	VIj	genome project	0	n.d.	(trace)
*Drosophila nasutoides*	Dna	VIj	this study	0	n.d.	AM050265

^a ^Abbreviations: I, Fungi; II, Mycetozoa; III, Ctenophora; IV, Vertebrata (a, Mammalia; b, Aves; c, Amphibia; d, Teleostei); V, Urochordata; VI, Arthropoda (a, Chelicerata; b, Collembola; c, Thysanura; d, Odonata; e, Dermaptera; f, Hemiptera; g, Hymenoptera; h, Coleoptera; i Lepidopdera; j, Diptera)

^b ^inside of *Su(var)3-9*-specific sequences; *two gene copies in one species; n.d., not determined.

### Alignment and phylogenetic analysis

The sampled Su(var)3-9 proteins were aligned using the ClustalW option of MacVector 7.2 (Accelrys). The programs MrBayes3.1 [45], Tree-Puzzle5.2 [46], PAUP4.0b10 [47], PHYLIB 3.63 [48] and Weighbor [49] were used for phylogenetic analyses. Tree constructions were performed through the bayesian inference (BI) method by MrBayes using the mixed substitution model and four gamma rate categories, 500,000 replicates (every 100 th was saved) and a burn-in of 2000 resulting in 6000 trees. For a maximum likelihood (ML) analysis, we used quartet puzzling by Tree-Puzzle with 10,000 puzzling steps, the WAG substitution model and assuming rate heterogeneity with invariants plus eight gamma rate categories. A maximum parsimony (MP) analysis was done by heuristic bootstrapping (1000 steps) using PAUP and the branch-swapping algorithm tree-bisection-reconnection (TBR). Finally, a weighted neighbor joining analysis was calculated by Protdist (PHYLIB) and Weighbor using the JTT substitution model.

### Analysis of amino acid substitutions in mutants

Alignments of sampled H3K9 methyltransferases were used to infer homologous residues to amino acid substitutions found in *D. melanogaster Su(var)3-9 *mutants. According to Kondrashov et al. [50], we identified compensated pathogenic deviations (CPD) in other HMTases. CPDs are amino acid substitutions that, at this site, would show a mutant phenotype in *D. melanogaster*. To identify CPDs, both *D. melanogaster *wild type and mutant Su(var)3-9 proteins were modelled according to known H3K9 HMTase crystal structures using the Swiss Model server [51]. We determined all amino acid residues that directly interact with a CPD residue, requiring that the distance between their closest atoms does not exceed 4 Å in wild type or mutant. We used multiple alignment to check whether an amino acid that is deleterious in flies can co-occur with *D. melanogaster *amino acids at this interacting sites. If the co-occurrence was never observed, and if all wild type proteins that carry the CPD also carry such a particular amino acid at the second site, we hypothesized that this second-site substitution is compensatory.

## Authors' contributions

VK conceived the study, designed the experiments, participated in sequencing, performed most of the bioinformatic analysis and wrote the manuscript. AF, PF and IP sequenced most of the arthropod *Su(var)3-9 *genes and carried out preliminary analyses of the sequence data. HS supervised the work. All authors participated in editing of the manuscript. All authors read and approved the final manuscript.

**Table 2:** Classification of *D. melanogaster Su(var)3-9 *mutants, based on structure modelling and comparison with orthologous proteins.

*Allele*	*Amino acid sub-stitution*	*Phenotype [6]*		*Conserved in*	*Occurrence of the substitution and compensation [50]; Function of residue*
		* in vivo (PEV)*	*in vitro (HMTase)*		

Class I – absolute conserved residues					
2,7	C427Y	null	null	all HMTases	zinc coordination [37]
21	E455V	Hypo-morph	null	all HMTases	-
31	E455K			all HMTases	intramolecular charge interaction [33]
3	R469C		null	all HMTases	intramolecular charge interaction [33]
27	G491R		null	all HMTases	Adomet (substrate) binding [32]
12,23	V492E			all HMTases	intramolecular hydrophobic interaction [33]
4,5	G509E	null	null	all HMTases	structural residue glycine [33]

**Class II – compensable deviated residues (CPDs)**					

30	A437T		null	Su(var)3-9, dim5	T in all beetles, compensated by L435G/S
9	C505Y		null	Su(var)3-9, G9a, SUVH, SETDB	L in *Araneus diadematus*, compensated by V585A
34	E506K			Su(var)3-9, G9a, SUVH	K in *Acyrthosiphon pisum*, compensation unknown (many substitutions in contact area of the residue)
320	D536N			Su(var)3-9, dim5, G9a	N in *Dictyostelium discoideum*, compensated by Y537G and N538D
32	S562F	null	null	all HMTases	L in some G9a proteins, compensated by S599G
13	P571S	Hypo-morph	Hypo-morph	Su(var)3-9 (Drosophila), SUVH, dim5	S in *Mus musculus *Suv39h2 and in mosquitos, compensated by C572V and H583R; A ribose hydroxyl of the methyltransferase reaction end product AdoHcy interact with the side chain carboxyl of the homologous D202 in Neurospora dim-5 [32]
10	D601N	null	Hypo-morph	most of all HMTases	N in mosquitos, *Acyrthosiphon pisum *and in some other HMTases, compensation ambiguous; involved in formation of lysine (substrate) binding channel [32]
8	N607Y	null	Hypo-morph	Su(var)3-9 (Drosophila)	no substitution or residue not conserved
25	P611L	Hypo-morph	Hypo-morph	Su(var)3-9 (Drosophila)	no substitution or residue not conserved
319	S616L			Su(var)3-9 (Drosophila)	no substitution or residue not conserved
22	L634F	Hypo-morph	Hypo-morph	Su(var)3-9, dim5, SETDB	F in *Enallagma cyathigerum*, compensated by F635M

**Class III – Uncompensated gain of function alleles**					

ptn	R529S	Hyper-morph	Hyper-morph	Su(var)3-9	S in three fishes and in *Acyrthosiphon pisum*, local compensation is excluded; the side chain of the homologous L317 in Neurospora dim-5 is involved in HMTase activity [32]

## Supplementary Material

Additional File 1 Sequencing strategy. This file (PDF format) describes the sequencing stategy used for the Su(var)3-9 genes of the analyzed arthropod species. Each gene is represented by a line drawing. Click here for file

Additional File 2 Su(var)3-9 protein alignment. This file (PDF format) contains the alignment which was used for phylogenetic analysis.
Click here for file

Additional File 3 Primer table. This file (PDF format) is a complete list of the primers used for PCR and sequence analysis. Click here for file
