# Supplementary material for: The evolution of the histone methyltransferase gene Su(var)3-9 in metazoans includes a fusion with and a re-fission from a functionally unrelated gene
Source: BMC Evol Biol. 2006 Mar 2;6:18. doi: 10.1186/1471-2148-6-18 (PMC1435931; doi:10.1186/1471-2148-6-18)
Supplement: Additional File 1 — Sequencing strategy. This file (PDF format) describes the sequencing stategy used for the Su(var)3-9 genes of the analyzed arthropod species. Each gene is represented by a line drawing. [file 1471-2148-6-18-S1.pdf]

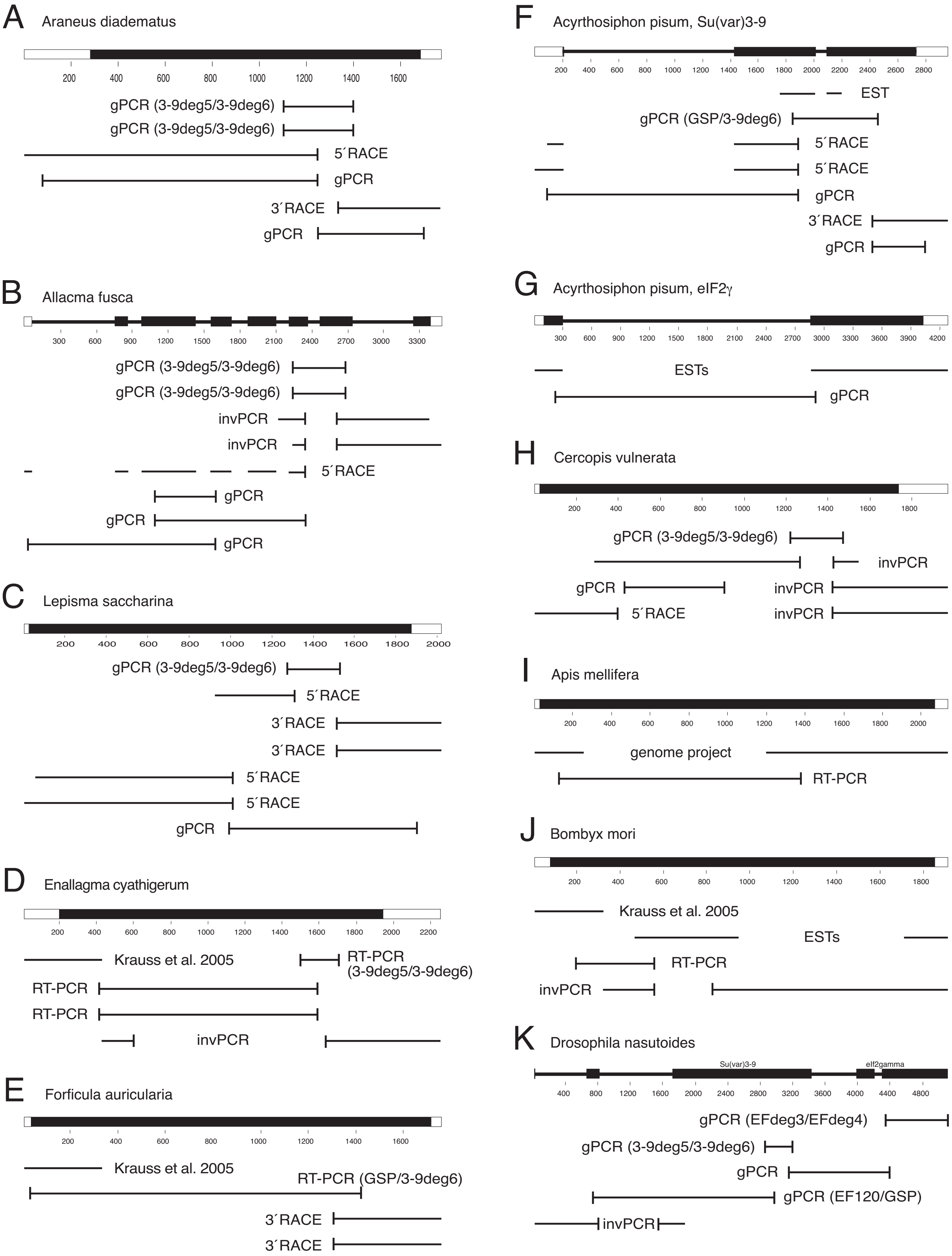

Supplementary Figure 1. Sequencing strategy used for the Su(var)3-9 genes of the analyzed arthropod species. Scaled sequences which were determined are shown. Coding parts are represented by black filled boxes. Untranslated transcribed regions are shown as empty boxes. Intronic and intergenic sequences are drawn as thick black lines. PCR fragments used for sequencing are shown beneath each structure outline, represented by thin lines. Primer positions are given by vertical hatches. Used degenerate oligonucleotide primers (see Material and Methods) are shown for every fragment. Names and sequences of gene specific primers (GSP) will be provided upon request. Further abbreviations used: RT-PCR, reverse-transcription polymerase chain reaction; RACE, rapid amplification of cDNA ends; gPCR, genomic polymerase chain reaction; invPCR, inverse (genomic) polymerase chain reaction; EST, expressed sequence tag.
