# Supplementary material for: The evolution of the histone methyltransferase gene Su(var)3-9 in metazoans includes a fusion with and a re-fission from a functionally unrelated gene
Source: BMC Evol Biol. 2006 Mar 2;6:18. doi: 10.1186/1471-2148-6-18 (PMC1435931; doi:10.1186/1471-2148-6-18)
Supplement: Additional File 2 — Su(var)3-9 protein alignment. This file (PDF format) contains the alignment which was used for phylogenetic analysis. [file 1471-2148-6-18-S2.pdf]

Supplementary Figure 2. Su(var)3-9 protein alignment used for phylogenetic analysis

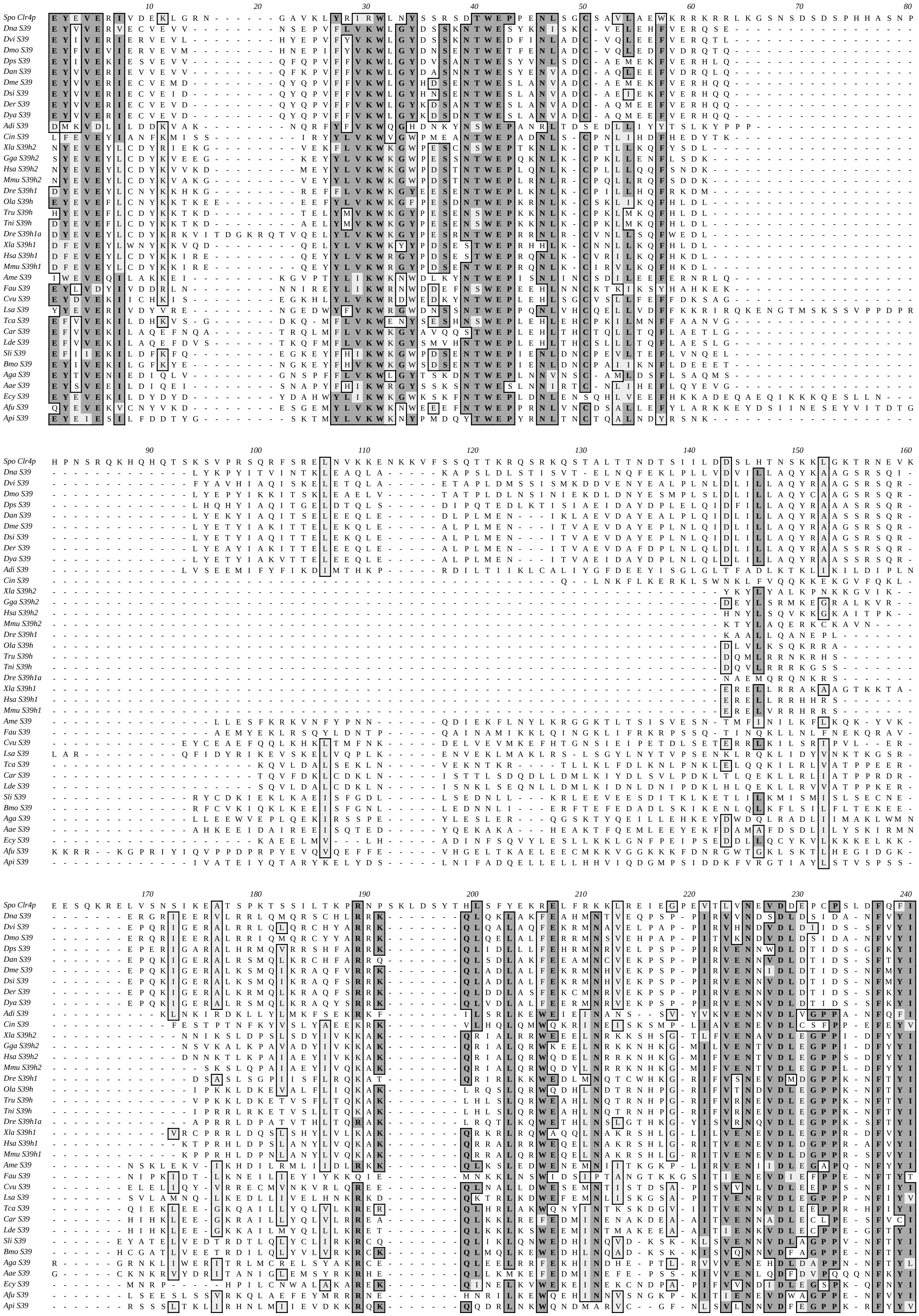

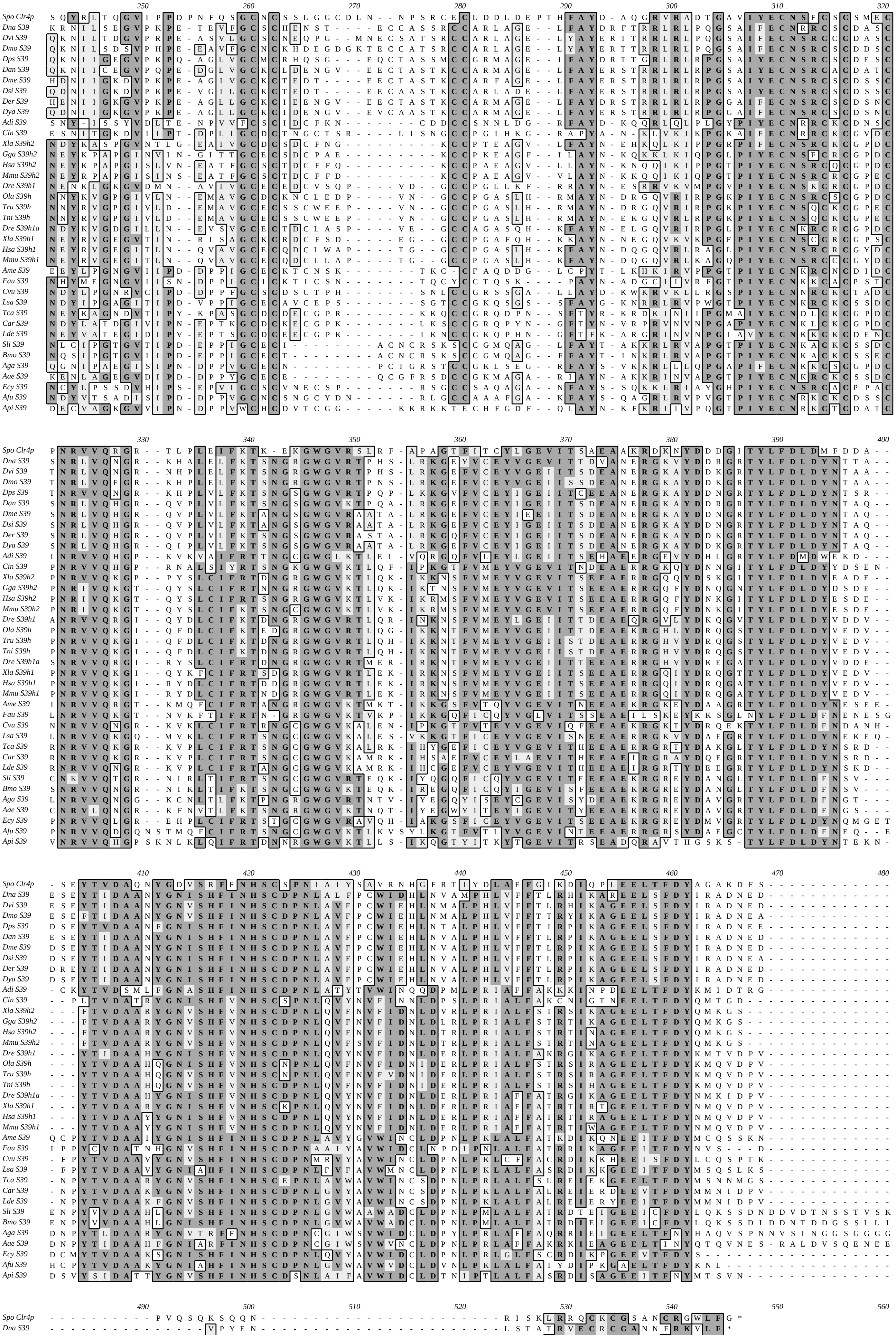
Spo Clr4p
Dna S39
Dvi S39
Dmo S39
Dps S39
Dan S39
Dme S39
Dsi S39
Der S39
Dya S39
Adi S39
Cin S39
Xla S39h2
Gga S39h2
Hsa S39h2
Mmu S39h2
Dre S39h1
Ola S39h
Tru S39h
Tni S39h
Dre S39h1a
Xla S39h1
Hsa S39h1
Mmu S39h1
Ame S39
Fau S39
Cvu S39
Lsa S39
Tca S39
Car S39
Lde S39
Sli S39
Bmo S39
Aga S39
Aae S39
Ecy S39
Afu S39
Api S39

```
Dvi S39      - - - - - - - - - - - - - - - V P Y E N - - - - - - - - - - - - - - - - - - - - - - - L S T A V R V E C R C G A A N C R K V L F *
Dmo S39      - - - - - - - - - - - - - - - V P Y E N - - - - - - - - - - - - - - - - - - - - - - - L S T A A R V E C R C G A P N C R K V L F *
Dps S39      - - - - - - - - - - - - - - - V P Y E N - - - - - - - - - - - - - - - - - - - - - - - L S T A A R V Q C R C G A A N C R K V L F *
Dan S39      - - - - - - - - - - - - - - - L P Y E N - - - - - - - - - - - - - - - - - - - - - - - L S T A V R V E C R C G A E N C R K V L F *
Dme S39      - - - - - - - - - - - - - - - V P Y E N - - - - - - - - - - - - - - - - - - - - - - - L S T A V R V E C R C G R D N C R K V L F *
Dsi S39      - - - - - - - - - - - - - - - V P Y E N - - - - - - - - - - - - - - - - - - - - - - - L S T A V R V E C R C G A D N C R K V L F *
Der S39      - - - - - - - - - - - - - - - L P Y E N - - - - - - - - - - - - - - - - - - - - - - - L S T A V R V E C R C G A D N C R K V L F *
Dya S39      - - - - - - - - - - - - - - - L P Y E N - - - - - - - - - - - - - - - - - - - - - - - L S T A V R V E C R C G A D N C R K V L F *
Adi S39      - - - - - - - - - - - - K H G I P V P E - - - - - - - - - - - - - - - - - - - - - - - - - - D E R V P C K C N S K N C R K F L F *
Cin S39      - - - - - - - - - - - - - N T T D T T N - - - - - - - - - - - - - - - - - - - - - - - P S S I K R T R C L C A S P N C R E W L V *
Xla S39h2    - - - - - - - - G D L S T D S I D M S P - - - - - - - - - - - - - - - - - - - - - - - A K K R V R I A C R C G A A T C R G F L N *
Gga S39h2    - - - - - - - - I D L T S D S A D G L S S - - - - - - - - - - - - - - - - - - - - - - S R K R I R T V C K C G A V C C R G Y L N *
Hsa S39h2    - - - - - - - - G D I S S D S I D H S P - - - - - - - - - - - - - - - - - - - - - - - A K K R V R T V C K C G A V T C R G Y L N *
Mmu S39h2    - - - - - - - - G E A S S D S I D H S P - - - - - - - - - - - - - - - - - - - - - - - A K K R V R T Q C K C G A E T C R G Y L N *
Dre S39h1    - - D A E S T K M D L D F S R A G I E G S - - - - - - - - - - - - - - - - - - - - - - P I K R V H M E C K C G V R N C R K Y L F *
Ola S39h     - - D T E S T K M D S S F S L A G L P G S - - - - - - - - - - - - - - - - - - - - - - P K K R V R V E C R C G S E L C R K Y L F *
Tru S39h     - - D T E S T K M D S S F G L A G L T S S - - - - - - - - - - - - - - - - - - - - - - P K K R I R V E C R C G S D S C R K Y L F *
Tni S39h     - - D T E S T K M D S S F S L A G L T S S - - - - - - - - - - - - - - - - - - - - - - P K K R I R V E C R C G S D S C R K Y L F *
Dre S39h1a   - - D A E S T K M D T N F G V M G L P G S - - - - - - - - - - - - - - - - - - - - - - P K K R M R V E C K C G V A T C R K Y L F *
Xla S39h1    - - D V E S S K M D S N F G I A G L P A S - - - - - - - - - - - - - - - - - - - - - - P K K R V R V E C K C G V S S C R K Y L F *
Hsa S39h1    - - D M E S T R M D S N F G L A G L P G S - - - - - - - - - - - - - - - - - - - - - - P K K R V R I E C K C G T E S C R K Y L F *
Mmu S39h1    - - D M E S T R M D S N F G L A G L P G S - - - - - - - - - - - - - - - - - - - - - - P K K R V R I E C K C G T T A C R K Y L F *
Ame S39      - - S E N S I M Q R A S M K E N L N V Y P E F Q E N V Q L - - - - - - - C S E I S E S Y S S N N K T L C K C D A Q N C R R Y L F *
Fau S39      - - S F G D T P K R T A P K S P L R M K S P Y G S - - - - - - - - - - - - - - - - - - - S K K N R I P C L C S A D K C R R V L F *
Cvu S39      - - S - - - K Q K N K I I P K T D G E R N S F K M H - - - - - - - - - - - - - - - - - - - - - - - - - C K C G S K N C R K Y Y F *
Lsa S39      - - S D L N S S R F K L S M Q D T M E E G T T D I H E G - - - - - - - - - - - - - - - D E I K G R I Q C K C K S T S C R K Y L F *
Tca S39      - - P M N T P E K S R P K L Q T P E K N E I M N G K L - - - - - - - - - - - - - - - - - - L P G T S I C K C A A D S C R R Y L F G *
Car S39      - - V P T T P E K S R - F L H T P D K N Q V I Q - - - - - - - - - - - - - - - - - - - - - - N G R N I C K C E A D S C R R Y L F *
Lde S39      - - V P T T P E K S R - F L H T P D K N Q V I Q - - - - - - - - - - - - - - - - - - - - - - N G R S I C K C E A D S C R R Y L F *
Sli S39      E G S V D D I P T G S S C D D I P S G S E A T A A I A P V S P V K S R F E I Q Q Q N R A M L R N L T E C K C G A L K C R K Y L F *
Bmo S39      K S S Y N D D I S L S N V S E I G T A S - - - - - - S P A S P L K S R F E I Q Q Q N M A M L R N R T E C K C G A M K C R K Y L F *
Aga S39      P P M D G D S S T G G V V E E K P A E N - - - - - - - - - - G D K A - - T T A N G S V R N T K G V T E C L C G S A N C R K F I F *
Aae S39      K Q P E G D - - - G G P G R G K P D P - - - - - - - - - - - - - - - - - - - - - - - - - - - E N L T E C R C G A A N C M K Y V F *
Ecy S39      - - - - - - P H Q G C G K A N K M S R A R - - - - - - - - - - - - - - - - - - - - - - - - - - - G T Q C R C G A K S C R K V F M *
Afu S39      - - V E E R V S K G L G N T S L D S E E G E K K I - - - - - - - - - - - - - - - - - - - - - K F N K E C L C G T K D C R K F L F *
Api S39      - - - - - - - - - - - - - - - - - N E N - - - - - - - - - - - - - - - - - - - - - - - - - - - R R I K C K C L S D N C R G Y L C *
```
