# Supplementary material for: The evolution of the histone methyltransferase gene Su(var)3-9 in metazoans includes a fusion with and a re-fission from a functionally unrelated gene
Source: BMC Evol Biol. 2006 Mar 2;6:18. doi: 10.1186/1471-2148-6-18 (PMC1435931; doi:10.1186/1471-2148-6-18)
Supplement: Additional File 3 — Primer table. This file (PDF format) is a complete list of the primers used for PCR and sequence analysis. [file 1471-2148-6-18-S3.pdf]

Supplementary Table 1. Primer used for PCR analysis

Species	Primer name	Sequence
Araneus diadematus	Adia3-9-1	GCACAAGTTCCAAGGTTTTTAGAC
	Adia3-9-2	CCCCAACCACATCCATTTG
	Adia3-9-3	TGCTGAAGAAAGAGGAGAAGTTTATG
	Adia3-9-4	CCTGCTATTTGCTGCTCTGTC
	Adia3-9-5	AATGGATGTGGTTGGGGTC
	Adia3-9-6	GTTTGCACACATAAAGAG
Allacma fusca	Kug3-9-1	CGCTCAGCCTCTTCAGTGTTTATG
	Kug3-9-2	TTGACACCCCAGCCACAGC
	Kug3-9-3	GCCCTTACACCGTAGACG
	Kug3-9-4	AAGAACCTGGTGGAAGAGCG
	Kug3-9-5	GCCTGTCCATCCTCGATTG
	Kug3-9-6	CACCTTTGGTTCCTGAAGAC
	Kug3-9-7	GATTCCTGATGACCCACC
	Kug3-9-8	GGTGTGTTGCATTGCCTCC
	Kug3-9-9	TTCTCAACTTCATACTGGTCTTC
	Kug3-9-10	GGAAAGCAAAGTAGATCCTG
Lepisma saccharina	Lsa3-9-1	TGAAGAGGCAGAGAGAAGAGGG
	Lsa3-9-2	AAGCCTTCACTCCCCACC
	Lsa3-9-3	GCACCACCCTATTCAAACAATC
	Lsa3-9-4	ACTGTTCCTTCTCGTTGTAATC
	Lsa3-9-5	GATTGTTTGAATAGGGTGGTGC
	Lsa3-9-6	GCTGAAGGACTGGGAGTTC
	Lsa3-9-7	GCGAAATCAGGTTCATCTCG
	Lsa3-9-8	GGAGATAAAACAAGGTTGGCAGTG
	Lsa3-9-9	AAGATTTATCAAAACTGGATGTAAC
	Lsa3-9-10	GTAGTGAAGGCTATTTCTGGAG
Enallagma cyathigerum	EcyEF3	GATTTGAAGGAAGCAGCG
	EcyEF4	AATGAGTTGGAGCGGAAC
	Ec3-9-1	GGTCGGACTTATCTATTTGATTTG
	Ec3-9-2	ACGCTTTTCAGCCTCTTCG
	Ec3-9-3	GATGGGAGATGCTGTAACAC
	Ec3-9-4	CATTCCTTTCTTGCCGAAG
Forficula auricularia	For5	AGGAGGCAATTTGGTATAGC
	For8	GGAGGCGAAGGTGTAAGC
	For12	GTTTAGTTATTACATCATCAGAAGC

Species	Primer name	Sequence
Acyrthosiphon pisum	Api1	CAAAAAAAGACAAAAACAACAAG
	Api2	GCCTTTGCCTGCCACAC
	Api3	TCAACGGGCTGTCACAC
	Api4	AAWCCACATACTCTTGCC
	Api5	GAGTCCGTCCGTCTGCC
	Api6	GAGTCCGTCCGTCTGCC
	Api7	GGTCATAATCCTCTACGG
	Api8	GCTCCCGTATGTATCATCG
	ApiEF1	GCGCACGGAAAATCAACTATTG
	ApiEF2	GACACAAAACAAGCAGGTCG
Cercopis vulnerata	Cer3-9-1	CCACAGCCATTTCTTGTTCTG
	Cer3-9-2	GACTTCAACGATGCCAATCAC
	Cer3-9-4	AAGACAGAACAGAACATCGCCAG
	Cer3-9-5	TTCACTTTCCCAATCTAACAACGC
	Cer3-9-8	GGTTGCTTTCTGGGTTAGG
Apis mellifera	Apis3	CATTATCCCGTGAAGTTATCAGC
	Apis7	CATAATCCATCATCCTGAG
Bombyx mori	BomEF2	CAACCATTATGGCTTCG
	Bom3-9-1	GCAGACCATCCTTTCCATTTC
	Bom3-9-2	GCTCATTTGGCAACCTCTTAG
	Bom3-9-3	AGAAAGAGGAGCATTGTGG
	Bom3-9-4	TGGAGAAGAAATATGCTTTGAC
Drosophila nasutoides	nasEF1	ACTGCTCCTTTGCCTGACTC
	nasEF2	AGTTTGTTCAGGGCACCGTC
	nasEF3	CGAAATCTTGCTGGTCAAC
	nasSu1	ACAATACGACGGCGGAGAG
	nasSu2	GCTGGTCTTGAACAACTCC
	nasSu3	TCAAAACATAAACTACTGACG
	nasSu4	GAAATGGTGCTGAGGTC
	nasSu5	GAGCTTTTTGCGAATTG
	nasSu6	GTGTAATGTTTCTTTCCAACTCG
	nasSu7	GTTGTCAGCGATTTAGGGAG
